# Validation of the InnoWell Platform: Protocol for a Clinical Trial

**DOI:** 10.2196/13955

**Published:** 2019-05-31

**Authors:** Tracey A Davenport, Haley M LaMonica, Lisa Whittle, Amelia English, Frank Iorfino, Shane Cross, Ian B Hickie

**Affiliations:** 1 Brain and Mind Centre The University of Sydney Camperdown Australia; 2 InnoWell Pty Ltd Camperdown Australia

**Keywords:** clinical trial protocol, mental health, medical informatics, suicide

## Abstract

**Background:**

New electronic health technologies are being rapidly developed to improve the delivery of mental health care for both health professionals and consumers and better support self-management of care. We developed a Web-based platform (the InnoWell Platform) that supports the prevention, early intervention, treatment, and continuous monitoring of mental health and maintenance of well-being in people aged 2 years and older. The platform is a customizable digital tool kit that operates through existing service providers who utilize the system to provide their consumers with access to evidence-based assessments and feedback, intervention options, and outcome monitoring. It does this by collecting, storing, and reporting personal and health information back to consumers and their health professionals to promote collaborative care partnerships that aim to improve the management of mental ill health and maintenance of well-being

**Objective:**

The aim of this study was to describe the research protocol for a naturalistic prospective clinical trial wherein all consumers presenting for care to a traditional face-to-face or Web-based mental health service in which the InnoWell Platform is being offered as part of standard clinical care will be given the opportunity to use the platform.

**Methods:**

The Web-based platform is a configurable and customizable digital tool that assists in the assessment, monitoring and management of mental ill health, and maintenance of well-being. It does this by collecting, storing, and reporting health information back to the person and his or her clinician to enable transformation to person-centered care. The clinical trial will be conducted with individuals aged 2 years and older presenting to participating services for care, including persons from the veteran community, Aboriginal and Torres Strait Islander people, people from culturally and linguistically diverse backgrounds, the lesbian, gay, bisexual, transgender, and intersex community, and those from broader education and workforce sectors, as well as people with disabilities, lived experience of comorbidity, complex disorders, and suicidality.

**Results:**

Project Synergy was funded in June 2017, and data collection began in November 2018 at a youth mental health service. At the time of this publication, 5 additional services have also begun recruitment, including 4 youth mental health services and a veteran’s service. The first results are expected to be submitted in 2020 for publication.

**Conclusions:**

This clinical trial will promote access to comprehensive, high-quality mental health care to improve outcomes for consumers and health professionals. The data collected will be used to validate a clinical staging algorithm designed to match consumers with the right level of care and reduce the rate of suicidal thoughts and/or behaviors and suicide by suggesting pathways to care that are appropriate for the identified level of need, while simultaneously enabling a timely service response.

**Trial Registration:**

Australian New Zealand Clinical Trial Registry ACTRN12618001676202; https://www.anzctr.org.au/Trial/Registration/TrialReview.aspx?id=374632 (Archived by WebCite at http://www.webcitation.org/78TOi5jwl)

**International Registered Report Identifier (IRRID):**

DERR1-10.2196/13955

## Introduction

### Background

As we move further into the twenty-first century, major revolutions in technology are transforming the way we live our lives—from the way we socialize, work, access information, and receive services. The mental health sector, like many others, is undergoing immense change in the face of this revolution, whereby new technologies are being developed at a rapid pace, many of which challenge the way people receive and manage their health care [1]. This is evident in the dramatic increase in the number of mobile apps, internet-based resources, and platforms that target mental health problems [2]. Although it remains a major priority to develop and assess effective Web-based interventions, an equally important task is to determine how these technologies can effectively integrate with existing health services in ways that improve the delivery of mental health care for the service, consumers’ health professionals, and presenting consumers [3].

The Australian Federal Government initiated the National Mental Health Strategy in 1992 [4], promoted by 5-yearly government plans outlining priority areas for investment and reform [5-9]. Despite the efforts of the last 25 years, the most recent report from the National Mental Health Commission (NMHC) continues to highlight fundamental shortcomings within the mental health system, including persistent stigma, poor experiences of care by individuals with lived experience, families and support people, delays in service provision, fragmented services, and inefficient and ineffective use of resources [10]. The NMHC review emphasizes the key concepts of person-centered care, personalized care options, regionalization of mental health services and suicide prevention, adoption of new technologies, and systematic evaluation to drive future investments [11]. In relation to the latter, the Fifth National Mental Health and Suicide Prevention Plan now includes 24 national key performance indicators that can be reported on to track the progress of the plan [9], including prevalence of mental illness, changes in mental health consumers’ clinical outcomes, rates of employment and social, community, and family participation among individuals with mental illness, and rates of suicide. Technology is increasingly recognized as a way to support and drive mental health service reform. Electronic health (eHealth) is broadly defined by the World Health Organization as the use of information and communication technologies for health-related purposes, such as service delivery [12]. Various models of eHealth services have been shown to be successful, including stand-alone systems for symptom prevention and self-help, consumer-assisted care, such as peer support, virtual clinics offering professional care, and stepped-care systems for integrated care [13]. The Australian Government published its “E-mental health strategy for Australia” in 2012 to promote its broader mental health reform agenda, emphasizing the ability of eHealth apps, etools, and platforms to deliver evidence-based interventions on the Web as a complement to traditional face-to-face services to improve health outcomes [14]. Importantly, eHealth services can improve access to mental health care by overcoming issues of distance, cost, and stigma.

### Stage-Based Stepped Care

In addition to revolutions in technology to support service delivery, there have also been advances in service models that aim to match consumers to the right level of care (ie, right care, right time). Clinical staging models are commonly employed in medical settings for this purpose, and they have more recently been implemented in clinical psychiatry [15]. Staging models consider the spectrum of mental ill health, and they aim to place consumers on that continuum, from those with risk factors with symptoms or impairment through to those with persistent and recurrent syndromes [15]. The emphasis is then on matching consumers at various stages to interventions that are appropriate to that stage [16]. As highlighted in Textbox 1, a staged-based stepped-care model combines the principles of clinical staging with the objectives of stepped care, including the use of low-intensity interventions for those at early stages and offering more intensive interventions to consumers at higher stages, while monitoring outcomes to increase or decrease service intensity as needs change [3].

### Suicide Prevention

The Zero Suicide in Health Care International Declaration was developed collaboratively by international leaders in mental health and addiction services with the aim of making suicide a “never event” [17]. This global initiative includes crucial recommendations and targets to promote suicide prevention worldwide, including fostering a safety-oriented culture to reduce the rates of suicide for consumers under care, employing an evidence-based approach to enhancing routine care and driving system changes that will result in improved outcomes and better care for those identified as being at risk, investing in training for health professionals aimed at improving identification, assessment, and management of suicidal thoughts and/or behaviors and risk factors thereof (eg, chronic pain, substance misuse), and systematically identifying, assessing, and monitoring suicidality in the broad consumer base for a service for the purposes of triaging individuals to the appropriate intensity of care. The abovementioned goals are synonymous with the core principles of the InnoWell Platform, which include increasing access to standardized, broad-based assessment, identifying and tracking consumer needs, matching those needs with personalized care options without having to wait for an appointment, enhancing the quality of the care provided to consumers, and supporting and guiding health professionals at all levels of experience to foster skill and professional development. Furthermore, the platform’s suicide escalation protocol is specifically designed to facilitate the detection of suicidal thoughts and/or behaviors and suggest pathways to care that are appropriate for the identified level of need, while simultaneously enabling a timely response from health professionals for those consumers reporting high suicidality [18]. Suicide prevention was highlighted as one of the priority areas in the Fifth National Mental Health and Suicide Prevention Plan [9]. Government agencies, service providers, and community agencies have all been identified as being key to reducing suicide rates and improving health outcomes. Despite efforts to improve suicide prevention in Australia, there has been no reduction in suicide rates over the past decade [19].

**Key features of stage-based stepped care.**

- Provide broad and holistic initial screening, followed by more targeted mental health assessment for those who endorse screening questions
- The intensity of the intervention should be matched to the consumer’s level of need as determined by clinical stage
- Provide parallel interventions for risk factors associated with poor outcomes (eg, unemployment, alcohol, and/or other substance misuse)
- Employ proactive monitoring of treatment progress and outcomes

### The InnoWell Platform

Through a process of participatory design with lived experience, health professionals, and service staff (including administration and management), we have developed a Web-based platform (the InnoWell Platform) that supports the prevention, early intervention, treatment, and continuous monitoring of mental ill health and maintenance of well-being in people aged 2 years and older [20]. The platform is a customizable digital tool kit that operates through existing service providers who utilize the system to provide their consumers with access to evidence-based assessments and feedback, intervention options, and outcome monitoring. It does this by collecting, storing, and reporting personal and health information back to consumers and their health professionals to promote collaborative care partnerships that aim to improve the management of mental ill health and maintenance of well-being. This platform uses multiple sources of information to develop a comprehensive understanding of the consumers’ needs and track their progress over time. This primarily involves Web-based self-reported psychometric measures from both consumers and their health professionals, as well as objective behavioral data collected via third-party integrations. Textbox 2 highlights the functionality built into the InnoWell Platform.

Specifically, in relation to suicide prevention, the InnoWell Platform employs a suicide escalation protocol derived from previous work [18]. In the current platform, suicidal thoughts and/or behaviors are assessed using 2 self-report surveys: Suicidal Ideation Attributes Scale [21] and Columbia-Suicide Severity Rating Scale [22]. Suicidal ideation without plan or intent in the past month triggers a pop-up for the consumer in the platform, providing details for crisis support services. Self-report of suicidal ideation with a plan and/or intent and/or a suicide attempt within the past 3 months triggers both the pop-up for the consumer and sends a notification to the consumer’s health professional, requiring a clinical care action (eg, telephone contact, action safety plan).

### Primary Objective

The primary objective of this clinical trial (Australian New Zealand Clinical Trial Registry ACTRN12618001676202) is to validate the algorithms used to derive stage-based stepped care on data collected by the platform and previously published staging criteria [15]. To do this, 4 independent ratings of stage-based stepped care will be compared: (1) platform generated, (2) health professional, (3) multidisciplinary team via consensus, and (4) expert-clinician reference group (comprising 3 or more academic health professionals with “area of research expertise” in stage-based stepped care for mental ill health). This expert group will review the clinical data of a subset of randomly generated consumers and independently allocate clinical stage.

### Secondary Objective

In accordance with the Fifth National Mental Health and Suicide Prevention Plan [9], the secondary objective of this clinical trial is to reduce the rate of suicidal thoughts and/or behaviors by promoting access to comprehensive services and reducing barriers to care, as well as improving service quality and evidence-based clinical interventions. It is hypothesized that use of the platform within traditional mental health services will facilitate earlier identification and rapid service response (eg, earlier appointment with a health professional) to high levels of suicidal thoughts and/or behaviors.

**InnoWell Platform functionality.**

- Multidimensional assessment across a range of domains (eg, sleep, anxiety, mood, physical health)
- Suicidal thoughts and/or behaviors and at-risk mental state identification and subsequent escalation to required intervention
- Dashboard of results across the range of biopsychosocial domains
- Algorithms to determine severity of needs across these biopsychosocial domains
- Algorithms to allocate clinical stage and suggested levels of care
- Real-time data tracking and interactive progress report
- Shared care planning between treating health professional and consumer
- Video-visit with a health professional
- Support person input and health information sharing
- Coordination of care across multi-disciplinary services
- Aggregate service performance indicator dashboard

## Methods

### Study Design

This paper employs a naturalistic prospective clinical trial design wherein all consumers presenting for care to a service utilizing the InnoWell Platform as part of their standard clinical care will be offered the opportunity to use the platform.

### Trial Site and Participating Centers

The InnoWell Platform is a Web-based app, and all data are collected electronically. As the sponsor and locality of the research team, the University of Sydney is considered the physical trial site. At the time of this publication, participants were being recruited from the following participating centers: headspace services (Camperdown, Coffs Harbour, Lismore, and Port Macquarie in New South Wales, and Edinburgh North in South Australia) and Open Arms—Veterans and Families Counselling in Surry Hills, New South Wales. Future participating centers may include services for children and their families, adult staged-care services, older persons mental health, general practice, as well as Aboriginal and Torres Strait Islander designed and controlled services.

### Participants

#### Eligibility Criteria

All consumers aged 2 years and older presenting for care to a traditional face-to-face or Web-based mental health service utilizing the platform as part of their standard clinical care will be eligible to participate in the clinical trial. This will include persons from the following populations: children and their families, young people, adults, older adults, the Veteran community, Aboriginal and Torres Strait Islander peoples, people from culturally and linguistically diverse backgrounds, the lesbian, gay, bisexual, transgender, and intersex community, and those from broader education and workforce sectors, as well as persons with disabilities, lived experience of comorbidity (including alcohol or other substance misuse), complex disorders, and/or suicidality. Owing to the nature of its research design, this clinical trial does not have defined exclusion criteria.

#### Sample Size

The clinical trial does not have an upper or lower limit on the number of participants.

#### Screening Procedures

It will be standard clinical care for all consumers presenting to a traditional face-to-face or Web-based mental health service utilizing the InnoWell Platform to be directed to the platform for assistance in assessment, management, and monitoring of their mental ill health and maintenance of well-being.

On contacting the service either by telephone (or short message service), on the Web (eg, email, chat), or in person, the consumer will be introduced to the clinical trial via an ethics-approved script. Although this script is generic and adaptable to different populations, settings, and services, it informs the consumer of what is involved in using the platform and notifies them that the data collected by the platform will be shared with their service provider to promote person-centered collaborative care and shared decision making. If the consumer is interested and responds “Yes,” they will receive a unique email invitation to sign up to the platform. When the participants receive the email invitation, they will be required to accept the invitation and be directed to “Sign up” by creating an account. If they do not sign up immediately, they will be sent a reminder via short message service. The participants will be required to review and accept both the “Privacy” and “Terms of use” within the platform before proceeding to a “Research data sharing” screen, which is where details about this clinical trial are contained.

#### Informed Consent

##### Opt Out Process

As part of the standard “Terms of use” of the platform, participants aged 14 years and older will be informed that their deidentified health data collected by the platform will be used for research purposes unless they “opt out.” If participants decide to “opt out,” they can indicate this by ticking a box. This action will be noted in the platform’s database, and it will automatically switch their access to the platform to read only, preventing them from entering any new data. If participants do not “opt out,” their deidentified data will be accessible by members of the research team. Importantly, participants will be able to edit their data sharing permissions at any time, such that any future data collected about them will not be accessible to researchers if, for example, they choose to withdraw from the clinical trial. The “opt out” consent approach described above is an efficient procedure without violating the option of providing choice. The approach taken considers a participant’s willingness rather than refusal to participate in the clinical trial and provides the necessary information to make an informed decision. Finally, the risk to the participants can be considered very low, as their data are deidentified (ie, all personal identifiers removed, including name, date of birth, and email address).

##### Parental Consent Requirements and Process

For children between the ages of 2 and 11 years, consent for the deidentified data to be used for research purposes will be provided solely by the parents/guardians. Child assent will not be sought. The parents/guardians will also serve as the “consumer” in these instances, answering the surveys in relation to the child. For consumers aged 12 and 13 years, parents/guardians will again be required to provide consent for the young person’s deidentified data to be used for research purposes. In addition, as per the standard process described above, the young person will be given the option to “opt out” of sharing his or her data through the standard “Terms of use” provided when participants access the platform. For deidentified data to be used for research purposes, both parents/guardians and the young person (if aged 12 or 13 years) need to have provided permission to do so (ie, if the parent consents but the young person “opts out,” the data will not be used and vice versa). Parents/guardians of children aged 13 years or younger will receive a standard introduction email to the platform sent by the service, which will include brief information regarding the research, as well as a link to further details, and a page where parents/guardians can indicate consent (or not) for the use of their young person’s data for research purposes. This parental consent is specifically related to the storage and use of their young person’s data for this clinical trial. Thus, if a parent/guardian chooses not to consent to the use of data for this clinical trial, there will be no impact on the young person’s standard clinical care; however, their access to the platform will be automatically switched to read only.

### The InnoWell Platform Description

**Figure 1:**
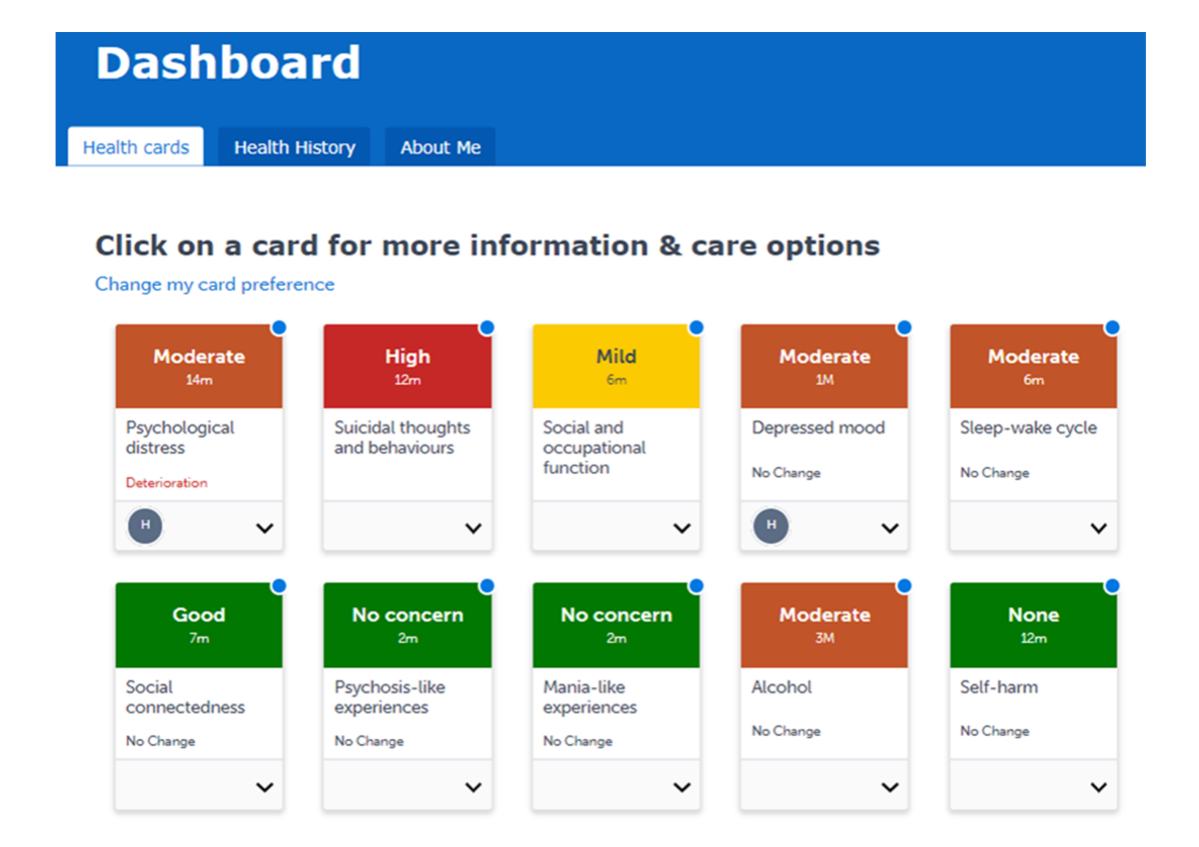
Sample personalized dashboard of results.

**Figure 2:**
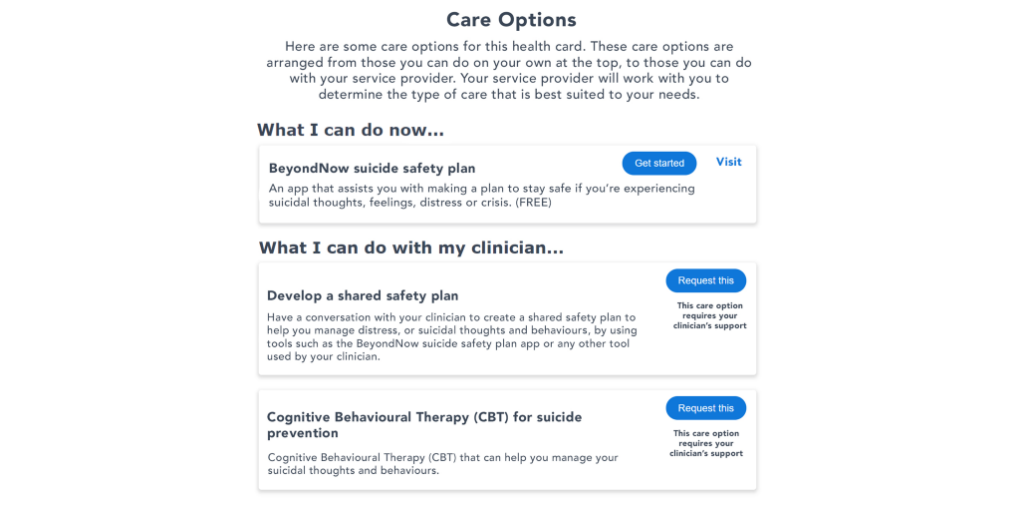
Sample clinical and nonclinical care options.

The InnoWell Platform is being embedded within traditional face-to-face and Web-based mental health services, and it is being offered to consumers presenting to those services as part of standard clinical care. It is a Web-based platform, and it can be accessed via traditional computing and mobile devices. As outlined in Textbox 2, the platform allows consumers to complete Web-based clinical assessments to understand their needs; explore their personalized dashboard of results, including current symptoms, level of functioning, and health history (see Figure 1); select from recommended care options (eg, fact sheets, apps, etools, and other Web-based systems) to support their mental health and well-being (see Figure 2); track their progress (in real time); share their dashboard and plan with their health professional(s) to support care.

### Participant Procedures

Participants will use the platform of their own accord as part of their standard clinical care with the mental health service. No other participation is required. All survey data are part of the functionality of the platform, which participants complete as part of standard clinical care through their service. The surveys provide assessment across a range of biopsychosocial domains, including psychological distress, suicidal thoughts and/or behaviors, social and occupational functioning, depressed mood, anxiety, sleep-wake cycle, social connectedness, psychosis-like experiences, mania-like experiences, alcohol use, tobacco use, self-harm, physical health, posttraumatic stress, eating behaviors, and body image. These domains, along with the associated surveys configured in the platform, are determined by services, in conjunction with the researchers to meet the needs of their consumers. Table 1 provides an example of the surveys that may be included across a sampling of biopsychosocial domains.

**Table 1:** Example domains and surveys.

Example domain	Survey
Anxiety	Overall Anxiety Severity and Impairment Scale [23] Generalized Anxiety Disorder Scale [24]
Depression	Quick Inventory of Depressive Symptomatology [25] Patient Health Questionnaire [26]
Functioning	Instrumental Activities of Daily Living and Physical Self-Maintenance Scale [27] Activities of Daily Living Index [28]
Trauma	Primary Care Posttraumatic Stress Disorder Screen for Diagnostic and Statistical Manual of Mental Disorders, Fifth Edition [29] Posttraumatic Stress Disorder Checklist [30]
Suicide behaviors	Columbia-Suicide Severity Rating Scale [22]

Completion of the surveys serves to populate a participant’s dashboard, which comprises health cards reflecting the scores (eg, mild, moderate, and high) generated from the participant’s responses (see Figure 1). The abbreviations under the scores reflect the time since the survey was last completed by the participant. For example, “1M” indicates the survey was completed one month earlier, and “14m” indicates the survey was completed 14 min earlier. By clicking on a health card in their dashboard, the participants can learn more about the biopsychosocial domain (eg, *This card tracks your symptoms of depression. Some signs of depression include feelings of worthlessness or losing interest in enjoyable activities*.). Links to psychoeducational resources are also available for further details about symptoms and treatment options. Each health card also contains a range of different care options, commonly known as interventions, to help the participant in managing a particular area of health. Care options are divided into 2 types: clinical and nonclinical (Figure 2). Clinical care options require a health professional’s involvement, such as individual therapy and group therapy. In contrast, a participant can immediately access and begin using nonclinical care options, such as apps, etools, or other systems, without the support of a health professional.

**Figure 3:**
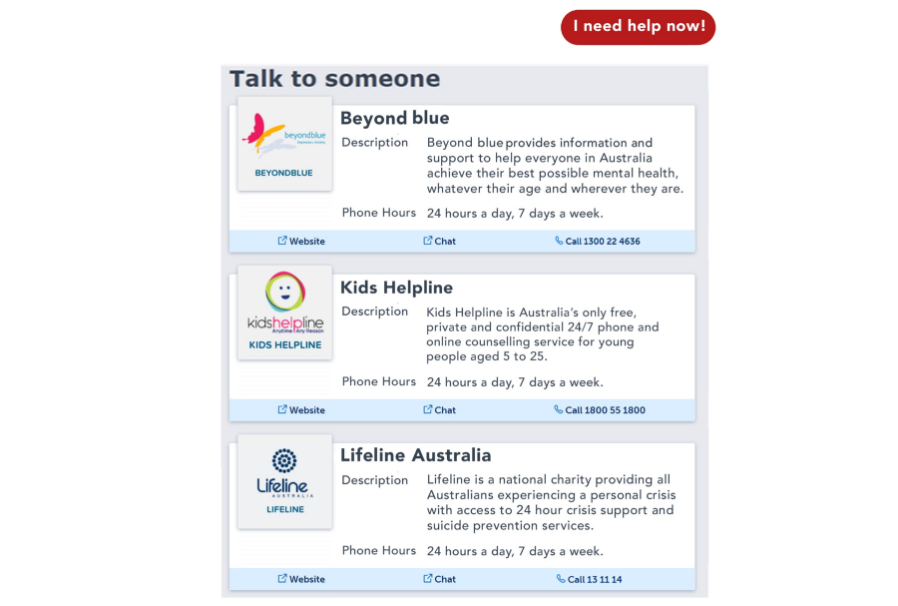
The “I need help now” button.

Given the emphasis on suicide prevention, the InnoWell Platform has been built to include immediate access to crisis support options. At any time when engaging with the platform, a participant has access to the “I need help now” button (see Figure 3), which provides contact details for crisis services (eg, Lifeline, Beyond Blue, and Kids Helpline). As described previously, these support options also appear as a pop-up directly after the participant scores low, moderate, or high on the suicidal thoughts and/or behaviors health card. When a participant scores high on this health card, a notification of suicide risk is also triggered for the treating health professional requiring clinical action.

### Configuration

The InnoWell Platform is highly configurable, allowing each service to adapt the technology to the needs of its consumer base. Although there is a set of core biopsychosocial domains that all services will be encouraged to assess (ie, psychological distress, suicidal thoughts and/or behaviors, psychosis-like experiences, mania-like experiences, social and occupational functioning, self-harm, tobacco use, alcohol use, social connectedness, depression, anxiety, and posttraumatic stress), the measures used to evaluate these areas can be tailored to the service. Similarly, the algorithms used to derive the thresholds for each domain will be based on the most appropriate psychometrics for that population (eg, young people, veterans, and older adults). As the platform is implemented across multiple health services and accumulates more data, these algorithms will become digitally smart.

### Education and Training Requirements

As stated previously, the implementation of clinical staging models is relatively new to clinical psychiatry [15], and therefore the education and training requirements of health professionals will need to be scoped to ensure consistency of application in each health service. As required, the training will cover the theoretical and scientific underpinnings of the model, the clinical assessment requirements, detailed criteria used to assign stage-based stepped care, the application of the model to subsyndromal, prodromal, or mixed syndromes, and the clinical utility of the model for the purposes of planning, implementing, and monitoring treatment. The method of training delivery may vary from service to service to ensure access and broad distribution.

### Outcomes

#### Primary Outcome

The platform-generated assignment of stage-based stepped care derived from an algorithm calculated from data collected by the platform will be compared with that allocated by the health professional working directly with the consumer, the within-service multidisciplinary consensus team, as well as by an independent expert-clinician reference group, all of which will rely on previous published clinical staging criteria [15]. For example, to meet criteria for Stage 2 or higher for depression, the disorder needs to have features indicative of more severe disorders, including psychomotor retardation, agitation, impaired cognitive function, severe circadian dysfunction, psychotic features, brief hypomanic periods, severe neurovegetative changes, or severe suicidality. Although a clinician will assess these features as part of standard clinical care to assign clinical stage, the platform will rely on the scores on several health cards (ie, depression, mania-like experiences, psychotic-like experiences, alcohol use, and eating behaviors and body image) for this purpose. The primary outcome will be the reliability of the ratings across these 4 methods of assigning clinical stage as assessed using Cohen kappa coefficient.

#### Secondary Outcome

**Figure 4:**
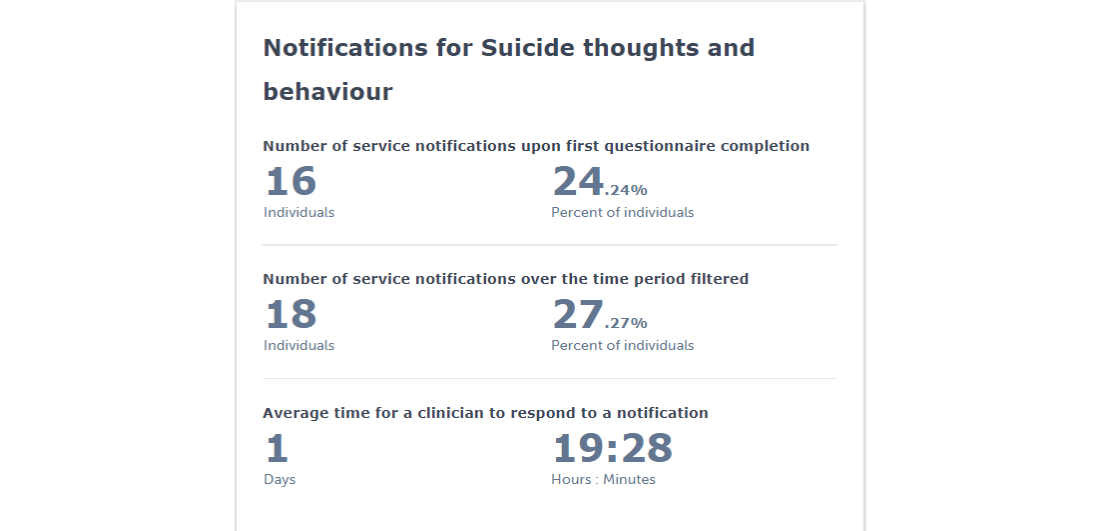
Hypothetical service-level performance data related to safety.

Service-level performance data regarding improvements in safety and clinical quality will be derived from the platform, with an emphasis on identification and rapid response to consumers endorsing suicidal thoughts and/or behaviors (see Figure 4). Outcomes will include the percentage of consumers endorsing suicidal thoughts and/or behaviors across 4 severity levels (ie, none, low, medium, and high) at service entry, the number of suicide escalations (ie, notifications to health professionals) for a specific period and during an entire episode of care, and time between suicide escalation and an action by a health professional (eg, call to consumer, schedule follow-up appointment, and contact emergency services).

### Data Collection, Management, and Security

All collected data are stored in the platform database that resides in the Google Cloud Platform (in Sydney, New South Wales) and the encrypted backup database that resides in the Amazon Web Services platform (in Sydney, New South Wales). The researchers’ access deidentified clinical trial data using straight-through digital processing methods such that a copy of clinical trial data (at any point in time) can be automatically moved from the platform database to a specified secure University of Sydney network server in one go.

This trial will be conducted in accordance with the Privacy Act of 1988 [31]. Upon consenting to participate in the clinical trial, participants will be assigned a unique identifier (Version 4 UUID), which is automatically generated by the platform and stored within its database. A Version 4 UUID is a universally unique identifier that is generated using random numbers. Importantly, the unique identifier will be used to identify a participant in all research studies of the InnoWell Platform should they be a consumer at more than 1 participating center. In addition, researchers will assign all participants with a Study ID number within deidentified clinical trial datasets. These Study ID numbers will be maintained separately from the data collection process and used for statistical analysis purposes only. All data are backed up hourly in a secure, long-term storage on Google’s Infrastructure.

### Data Analysis

Scientific validity will be evaluated using interrater reliability statistical methods, such as Cohen kappa coefficient, to quantify the degree of agreement within and among the 4 ratings of stage-based stepped care (ie, platform-generated, health professional allocated, in-service multidisciplinary consensus, and independent expert-clinician reference group). The clinical performance, including aspects of safety, of the platform will be evaluated using aggregate data derived from clinical rating scales completed by the consumer and health professional to measure and track health domain intensity, frequency, quality, and change over time, and aggregate service-level performance data, including safety and clinical quality (eg, time to first assessment, service accessibility, wait time for clinical intervention, service efficiency, and user satisfaction). Within- and between-group analyses using multivariate statistics (eg, multivariate analyses of variance, Kruskal-Wallis test) will be computed to evaluate differences in clinical outcomes among services and among population groups. In addition, reliable change and effect-size scores will be calculated to determine clinical improvement over time for consumer data and then aggregated for service-level outcome data. Analytical validity will be assessed using trends only (ie, descriptive statistics) as a means to determine user engagement and overall effectiveness of the technical performance of the platform.

### Ethics Approval

This trial has been approved by the Northern Sydney Local Health District Human Research Ethics Committee (NSLHD HREC), reference number HREC/17/HAWKE/480.

## Results

InnoWell is a for-profit-business that has built the technology platform underpinning this clinical trial. InnoWell is a joint venture between The University of Sydney and PricewaterhouseCoopers (PwC) Australia that aims to transform mental health through person-centered care. Beyond June 2020, InnoWell will support this clinical trial financially, as it represents the core research and development work necessary to continuously and iteratively evolve the platform. The first results are expected to be submitted for publication in 2020.

## Discussion

### Person-Centered Care

At its core, the InnoWell Platform is designed to promote person-centered health care and assist clinical practices that place consumers as equal partners in health care decision making. The platform is also intended to help minimize the variability in care provision between health professionals and services by supporting clinical judgement with data. In addition, the platform aims to maximize the use of available resources and minimize duplication of services and wastage of time for all users, including consumers, health professionals, and service staff.

### Feasibility of Implementation With Service

Before implementing this clinical trial, a thorough assessment of the feasibility of deploying the platform within each service (or participating center) is required [32,33]. This includes an understanding of the basic service attributes (eg, type of service, qualifications of health professionals, service location(s), and information and communication technology systems), as well as the identified needs of the organization that will be met by the platform. In other words, what problem(s) will the technology address? The fit of the InnoWell Platform to the values, priorities, and strategic plan for the service and the readiness of the service to adopt the technology also need to be examined. As the platform is customizable, it is necessary to identify in what way the technology needs to be modified or adapted to fit the service and its consumers. Finally, key to this process is the engagement of critical stakeholders who can drive the adoption of the platform, identify facilitators, mitigate barriers to implementation, and champion the technology among frontline staff whose practice will be most affected. The effective deployment of the platform will be supported by implementation officers embedded within the services. These people will ensure that research protocols are adhered to and report any adjustments that might be necessary; they will monitor, catalogue, and report on the progress of the implementation to key trial leads; they will collect data at the participating center, relating to facilitators or barriers of implementation from clinical and administrative staff; they will assist with preparation of and delivery of training to relevant personnel regarding information essential to site-specific implementation; they will ensure that the platform accurately and consistently reflects the research data; they will ensure that all participants’ safety and well-being are first priority, by liaising with technology specialists, the researchers, and the relevant participating center, and by following established ethical protocols.

### Ongoing Development of Functionality

Qualitative and quantitative data will be gathered from all users of the platform, including consumers, as well as health professionals and service staff, to inform the iterative redevelopment of the platform, including site-specific customization of any new required functionality, as well as (re)configuration of content, questionnaires, and algorithms. Furthermore, the data collected during iterative user-testing sessions (covered under site-specific ethics approvals from relevant Human Research Ethics Committees) will help to monitor, evaluate, and provide ongoing feedback about the quality, acceptability, and usability of the platform for continuous improvement of the platform.

### Future Research

This clinical trial will be run in parallel with a series of participating center-specific impact evaluation studies. These adjunctive studies will focus on gathering data through Web-based questionnaires, as well as workshops and semistructured interviews with service staff (ie, health professionals, managers, and administrators) relating to the impact of embedding the InnoWell Platform in their service. Topics to be covered will include digital readiness and competence, the impact of the platform on the service, the social return on investment, and the quality, usability, and acceptability of the platform. In addition, workshops will also offer the opportunity for researchers to introduce and provide brief training to service staff relating to new functionality that will be incorporated into the platform at quarterly intervals. Separate ethics approvals will be sought for these studies.

## Abbreviations

BMC: Brain and Mind Centre
eHealth: electronic health
NMHC: National Mental Health Commission
PwC: PricewaterhouseCoopers
